# Interesting Lawsuit Postponed

**Published:** 1881-09

**Authors:** 


Interesting Lawsuit Postponed.—The case of the State of Massachusetts vs. the Smith Manufacturing Company of Lee, Mass., was postponed from the 20th of this month to the next term of the superior court, owing to the sudden sickness of the state attorney. The case was one of unusual interest to manufacturers and to physicians. The company were indicted for occasioning malaria, and malarial fevers, by raising their dams and flooding new land. It will be a test case in the Massachusetts courts. The following named Connecticut physicians were summoned as experts upon the subject of malaria by the defense :—Professor Francis Bacon of New Haven, Dr. Rufus W. Griswold of Rocky Hill, Dr. F. D. Edgerton of Middletown, Dr. R. M. Griswold of North Manchester, and Dr. Wilson of Meriden.

Our readers will recognize the names of the Drs. Griswold among the medical experts from Connecticut as contributors of interesting articles to this Journal.

We are glad to perceive that there is a growing tendency in some of the courts, at least, to avail themselves of the *best* medical talent within their reach, when questions of a medico-legal character are to be adjudicated, and not to rely upon the opinions of those who may be most conveniently procurable on such occasions. H. L. B.

